# Personal

**Published:** 1912-08

**Authors:** 


### PERSONAL

(We would greatly appreciate the courtesy of contributions to this and other news departments.)

The editor notes that the new directory of the A. M. A. has acted as god-father, bestowing on him Dr. Bennett's front name. At various times, this name, most of the Christian names beginning in Al-, and a miscellaneous assortment of Old Testament names beginning with A, have been conferred by the kindly disposed. For a plain man, the initials are plenty and the editor would appreciate the courtesy of a correction by any possessing the directory, to A. L. Benedict, the full legal name.

---

On July 3, Dr. Ray H. Johnson of Buffalo, had a collision with another automobile and was cut in the head and face by broken glass. We are glad to note that he has fully recovered.

---

Dr. Mary B. Wetmore has sold the residence on lower Franklin St., Buffalo, so long occupied by her late husband and herself and has moved to the Matthewson, corner of Tracy and Morgan Sts.

---

Dr. Harold J. McDonald has been appointed District Physician for the first district of Buffalo at a salary of $500 and Dr.

Charles Leone for the sixth district at a salary of $400. The districts will soon be rearranged on an equal basis of population and at equal salaries—which we can state from personal experience in 1890, to be far from equable. The city physicians are almost the only city employees who are not paid a fairly adequate salary. At the lowest estimate, with full permission to engage in private practice, the salaries should be at least $1000. We are inclined to favor a service at double this sum, requiring exclusive attention.

---

Dr. A. E. Collins of Buffalo, had his automobile turn turtle on July 12. He was badly bruised and was at first thought to have sustained spinal injuries.

---

Dr. Frederick Seliheimer of Buffalo, returning from Lackawanna, on July 12, found a young eagle had flown into his automobile. This is the first time we have heard of this kind of a bird entering an automobile without an invitation.

---

The members of the Oneida County Medical Club held their third annual outing yesterday at the Spring House at Rexford Falls. After dinner, which was served by Landlord F. J. Mulligan, trap shooting and a game of base ball were enjoyed. Those present were: Dr. C. H. Baldwin, Dr. R. L. Baker, Dr. C. R. Hart, Dr. W. J. Schuyler, Dr. A. M. Johnston, Dr. T. C. Gifford, Dr. W. B. Roemer, Dr. W. H. Beattie, Dr. J. E. Gage, Dr. Andrew Sloane, Dr. F. S. DeLong, Dr. F. R. Ford and W. Bauer.

---

Dr. Frederick W. Bentley of North Tonawanda, has returned from a trip to Lake George.

---

Dr. Chas. Ferdinand Durand, late Proctologist of the German Hospital, Buffalo, has opened an office at 590 Huron Street, Toronto. His practice will be limited to diseases of the rectum.

---

Dr. Martin B. Tinker of Ithaca, has recovered from the effacts of an automobile accident.

---

Dr. James Wright Putnam sailed for Norway in July, he will return to Buffalo Sept. 1.

Dr. Jacob S. Otto has been appointed Assistant Prof. of Therapeutics; Dr. A. E. Woehnert Asst. Prof. of Clinical Medicine; Dr. W. W. Plummer Asst. Prof. of Orthopaedics and Radiology; Dr. Carl S. Tompkins Clinical Pathologist, in the Medical Dept. of the University of Buffalo. All are residents of Buffalo.

---

Drs. Peter W. Van Peyma, Edward J. Meyer, George F. Cott, Harry Mead, Henry Stadlinger of Buffalo, have been appointed a committee of the Alumni of the Medical Dept. of the University of Buffalo, to manage and increase a fund of $4000 raised by the Alumni to further medical education.

---

Dr. George J. Haller of Buffalo, has returned from Europe.

---

Dr. James P. Barr of Buffalo was recently thrown out of his automobile in a collision with a street car and had several ribs broken.

---

Dr. J. Walter Fitzgerald of Buffalo, is in Europe.

---

Dr. H. J. Benedict of Danbury, Conn., is motoring through the Adirondacks.

---

Dr. George W. T. Lewis of Buffalo, is at Lake Moraine, Hamilton, Ont.

